# Advancing Nanofiber Research: Assessing Nonsolvent Contributions to Structure Using Coaxial Electrospinning

**DOI:** 10.1021/acs.langmuir.3c01038

**Published:** 2023-06-30

**Authors:** Wanying Wei, Michael Wildy, Kai Xu, John Schossig, Xiao Hu, Dong Choon Hyun, Wenshuai Chen, Cheng Zhang, Ping Lu

**Affiliations:** † Department of Chemistry and Biochemistry, Rowan University, Glassboro, New Jersey 08028, United States; ‡ Department of Physics and Astronomy, Rowan University, Glassboro, New Jersey 08028, United States; § Department of Polymer Science and Engineering, Kyungpook National University, Daegu 41566, South Korea; ∥ Key Laboratory of Bio-based Material Science and Technology, Ministry of Education, Northeast Forestry University, Harbin 150040, China; ⊥ Chemistry Department, Long Island University (Post), Brookville, New York 11548, United States

## Abstract

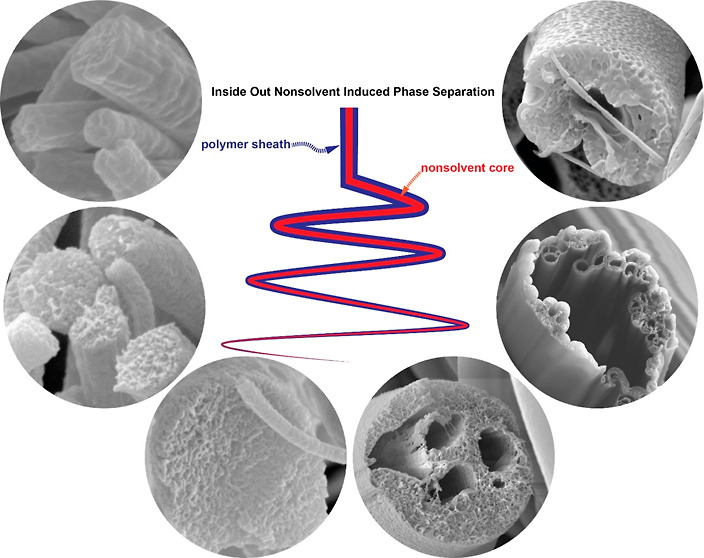

In this study, we explored the influence of molecular interactions and solvent evaporation kinetics on the formation of porous structures in electrospun nanofibers, utilizing polyacrylonitrile (PAN) and polystyrene (PS) as model polymers. The coaxial electrospinning technique was employed to control the injection of water and ethylene glycol (EG) as nonsolvents into polymer jets, demonstrating its potential as a powerful tool for manipulating phase separation processes and fabricating nanofibers with tailored properties. Our findings highlighted the critical role of intermolecular interactions between nonsolvents and polymers in governing phase separation and porous structure formation. Additionally, we observed that the size and polarity of nonsolvent molecules affected the phase separation process. Furthermore, solvent evaporation kinetics were found to significantly impact phase separation, as evidenced by less distinct porous structures when using a rapidly evaporating solvent like tetrahydrofuran (THF) instead of dimethylformamide (DMF). This work offers valuable insights into the intricate relationship between molecular interactions and solvent evaporation kinetics during electrospinning, providing guidance for researchers developing porous nanofibers with specific characteristics for various applications, including filtration, drug delivery, and tissue engineering.

## Introduction

Porous materials have attracted much interest because of their broad applicability in fields such as filtration, catalysis, and biomedicine.^1−3^ It is feasible to fine-tune their properties by employing systematic design principles and controlled fabrication techniques to cater to various applications.^4−7^ Important properties include the strength-to-density ratio, surface area-to-mass or volume ratio, thermal and chemical resistance, permeability, and insulation capacity. Advances in fabrication strategies and methodologies have expanded the range of available morphologies and properties of porous materials, providing the path for novel applications in areas such as drug delivery, tissue engineering, and regenerative medicine.^8,9^ To create porous materials, a variety of techniques have been developed, including electrochemical anodization, freeze-drying, sol–gel processes, self-assembly, three-dimensional (3D) printing, and atomic layer deposition.^10,11^ The majority of these methods rely on templating and phase separation, which are often combined with dissolution/extraction, etching, and high-temperature calcination or pyrolysis to form pores through the selective removal of sacrificial components.^12−16^

Electrospinning is an electrohydrodynamic atomization method. It has been widely recognized as a simple and versatile technique for generating continuous nanofibers and producing 3D constructs with hierarchical porosity by stacking nanofibers in an organized or random fashion.^17^ Electrospun nanofiber membranes can display two distinct pore types: interfiber and intrafiber pores.^18^ Interfiber pores, a signature feature of fibrous membranes, arise naturally in a nonwoven mat during electrospinning. The pore size and shape are adjustable by controlling electrospinning parameters or collection methods.^19^ Combining electrospinning with polymer phase separation techniques enables the fabrication of individual electrospun porous nanofibers.^20^ Among the various phase separation mechanisms, nonsolvent-induced phase separation (NIPS) emerged as a straightforward and practical method for generating electrospun porous nanofibers.^21^ One strategy involves incorporating a nonsolvent to form a polymer/solvent/nonsolvent ternary system. Such a system can induce porosity in electrospun fibers.^22^ A crucial aspect of this process is the choice of solvent/nonsolvent combination. It requires a difference in volatility to induce phase separation.^23^ As a result, the constant decrease in solvent/non-solvent ratio leads to a polymer-rich matrix and dispersed polymer-lean phases, culminating in porosity after ultimate evaporation.^24,25^ Researchers have demonstrated that highly porous polyacrylonitrile (PAN) and polyvinylidene fluoride (PVDF) fibers can be obtained by adding a small amount of water to a polymer/DMF solution before electrospinning.^26^ Alternatively, simply electrospinning the polymer solution into a nonsolvent bath before complete solvent evaporation can induce porosity.^27^ For example, Seo et al. found no porosity when electrospinning a control sample from a polycaprolactone/chloroform (PCL/CHL) solution, whereas they successfully fabricated porous PCL fibers by electrospinning the same solution into a water bath.^28^ Nonetheless, both approaches come with inherent disadvantages. First, adding a nonsolvent into the polymer solution may destabilize the mixture and reduce its spinnability. Second, controlling the solvent quantity in real-time during electrospinning remains a hurdle. Finally, when electrospinning polymers into a nonsolvent bath, loosely aggregated nanofibers are typically generated, which may be unsuitable for numerous applications that necessitate flat, nonwoven membranes, such as filtration.^21,29−32^

In this study, we present a unique approach to producing electrospun porous nanofibers by regulating the nonsolvent content within the liquid polymer jet using the coaxial electrospinning technique. The mixing of core and sheath solutions occurs solely within the liquid jet, thus maintaining the stability and spinnability of the polymer solution. Furthermore, nonsolvent-induced phase separation could be adjusted in real-time on individual nanofibers, allowing for a detailed examination of its impact on the generation of porosity in each nanofiber. Additionally, the diffusion process occurred from the inside out, as opposed to the slower nonsolvent bath diffusion,^33^ which proceeded from outside to inside and could be affected by the drying state of the nanofibers. Although coaxial electrospinning has been employed in the fabrication of core-sheath nanofibers^34^ and, in some cases, to facilitate the electrospinning of specific polymers using a non-electrospinnable fluid,^35,36^ to the best of our knowledge, no previous studies have reported its use to investigate in situ nonsolvent-induced phase separation during the electrospinning process. In our study, the core fluid contained a nonsolvent for polyacrylonitrile (PAN) and polystyrene (PS), resulting in the formation of pores at various scales in both polymer nanofibers. With the rapid core fluid injection, large hollow channels were also observed. Sometimes, the polymer sheath collapsed upon drying due to the presence of these sizable internal channels. The injection of nonsolvent into the polymer jet had a significant impact on the overall fiber structure, including size, shape, bead formation, and the spinnability of the polymer. This novel approach distinguishes our study from previous research in the field, offering new insights into the fabrication of electrospun porous nanofibers.

## Experimental Section

### Chemicals and Materials

Polyacrylonitrile (PAN) with a weight average molecular weight (*M*_w_) of 150,000 and Polystyrene (PS) with an *M*_w_ of 350,000 and a number molecular weight (*M*_n_) of approximately 170,000 were procured from Sigma-Aldrich and employed in the preparation of the outer fluid. Anhydrous *N*,*N*-dimethylformamide (≥99.9%, DMF, VWR), tetrahydrofuran (≥99.9%, THF, VWR), and ethylene glycol (≥99%, EG, Fisher Scientific) were utilized for dissolving the polymers or formulating the solvent/nonsolvent core fluid. No further purification was conducted on the received chemicals. Water, used in the experiments for preparing the nonsolvent core fluid, was subjected to purification via a Millipore Direct-Q 8 UV water purification system, resulting in a resistivity of 18.2 MΩ·cm at 25 °C.

### Exploration of Nonsolvent Influence on Polymer Nanofiber Structures via Coaxial Electrospinning

**Figure 1:**
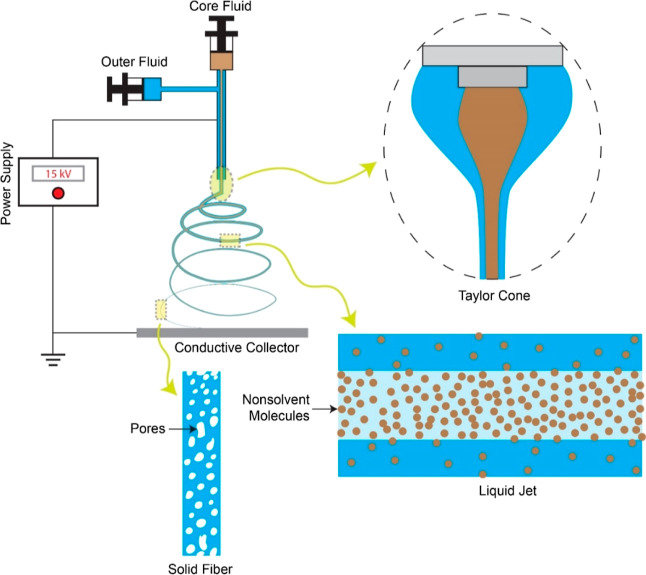
Schematic illustration showing the formation of porous nanofibers via core nonsolvent-induced polymer phase separation using the coaxial electrospinning technique.

The core nonsolvent’s impact on polymer nanofiber structures was examined using the coaxial electrospinning technique (Figure 1). In a standard experiment, an anhydrous DMF (or THF) solution containing 10% PAN (or 20% PS) was introduced at a rate of 1 mL/h into the outer needle of a metallic coaxial spinneret. Concurrently, a second DMF (or THF) solution with 5% water (or EG) was supplied to the inner needle. Independent control over the feed rates of the outer fluid (polymer solution) and core fluid (nonsolvent solution) was achieved using two programmable syringe pumps (Legato 110, KD Scientific). A high-voltage DC power supply (ES30P-5W, Gamma High Voltage Research) was connected to the stainless-steel coaxial spinneret. With the application of a 15 kV charge to the spinneret, a liquid jet comprising the core nonsolvent solution and the sheath polymer solution was ejected from the Taylor cone. The charged jet’s whipping and bending instability in the electric field led to rapid stretching, promoting the diffusion of the core nonsolvent into the polymer sheath, followed by nonsolvent-induced polymer phase separation. Subsequently, porous nanofibers were produced after the swift evaporation of the solvent and nonsolvent on a conductive collector positioned 25 cm below the needles’ tip. All electrospinning experiments were carried out at a temperature of 20 ± 2 °C and a relative humidity of 50 ± 3%. The laboratory’s central air conditioning system regulated the temperature, while an industrial-grade humidifier/dehumidifier controlled humidity. Prior to subsequent experiments and characterizations, the obtained nanofibers were dried in a vacuum oven for 24 h at room temperature.

### Characterization

High-resolution field-emission scanning electron microscopy (SEM, Apreo, FEI) was employed to examine the surface morphology and internal structure of the nanofibers. In order to reveal their internal structure, the nanofibers were first fractured in liquid nitrogen at −195.8 °C and subsequently vacuum dried. To enhance their electrical conductivity, all samples underwent sputter-coating with gold for a duration of 30–120 s, depending on the specific sample. Representative images of the samples were captured at an analytical working distance of 7 mm, employing an accelerating voltage of 10 kV and a beam current of 0.40 nA. Nanofiber size measurements were carried out using ImageJ software (NIH) based on the representative SEM images. Furthermore, the fiber size distribution was statistically analyzed with the aid of OriginPro software (OriginLab).

## Results and Discussion

### Nonsolvent-Induced Phase Separation in PAN Nanofibers

**Figure 2:**
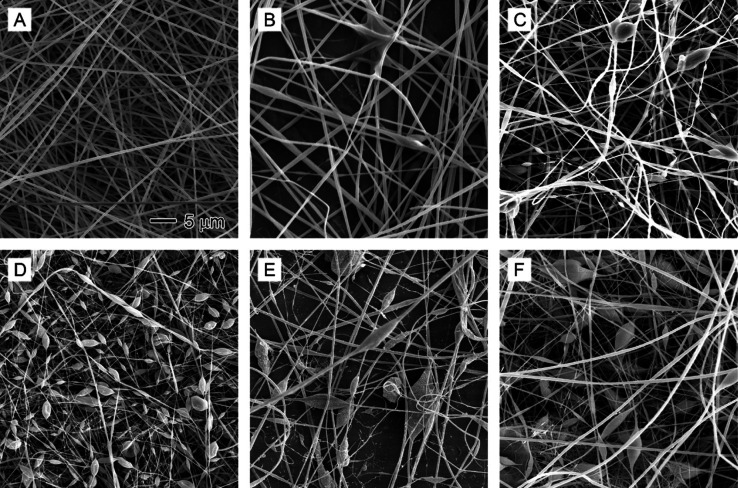
SEM images showing the effect of core nonsolvent injection rates on the formation of beads in polyacrylonitrile (PAN) nanofibers: (A) 0, (B) 0.1, (C) 0.3, (D) 0.5, (E) 0.7, and (F) 0.9 mL/h. The nonsolvent is a 1/20 (v/v) water/DMF solution. The sheath fluid is 10% PAN in DMF. The scale bar in (A) applies to all images.

Figure 1 presents a schematic illustration depicting the formation of porous nanofibers via core nonsolvent-induced polymer phase separation using the coaxial electrospinning technique. The polyacrylonitrile (PAN) nanofibers were fabricated utilizing this coaxial electrospinning setup. To facilitate the rapid diffusion of the nonsolvent into the PAN solution, the nonsolvent was dissolved in the same solvent used for PAN, i.e., dimethylformamide (DMF). The volume ratios between the nonsolvent and DMF were optimized to a 1:20 v: v ratio, which effectively minimized needle clogging across a wide range of core nonsolvent injection rates (0–1.0 mL/h). As both the core and sheath fluids employed DMF as the solvent, the mixing, diffusion, and penetration of the solvent, polymer, and nonsolvent were maximized within the liquid jet. This allowed for the formation of nonsolvent-rich domains and, consequently, the generation of pores inside the nanofibers. The fast evaporation of the solvent in the polymer-rich domains further facilitated pore formation, ultimately yielding porous PAN nanofibers, as illustrated in Figure 2.

Figure 2 presents SEM images of PAN nanofibers produced using coaxial electrospinning at various core nonsolvent (1/20 v/v water/DMF) injection rates. The sheath PAN solution was fed at a constant rate of 1 mL/h, which was optimized for all the experiments. Smooth and uniform PAN nanofibers were obtained in the absence of nonsolvent injection through the core needle (Figure 2A). Upon introducing nonsolvent at a rate of 0.1 mL/h, some regions of the nanofibers exhibited swelling (Figure 2B). As the nonsolvent injection rate increased from 0.3 to 0.5 mL/h, a greater number of beads formed within the nanofibers (Figure 2C,D). Despite this, the electrospinning process remained continuous, and needle clogging was not observed.

When the nonsolvent injection rate reached 0.7 mL/h, the electrospinning process became unstable, resulting in fewer fibers being produced. The collected nanofibers displayed significantly larger beads (Figure 2E). At a nonsolvent injection rate of 0.9 mL/h, the nonsolvent solution flowed from the inner needle, causing constant clogging and making the electrospinning process essentially unsustainable. Membranes containing a large number of beads, tiny nanofibers, and nanofibril mesh were obtained (Figure 2F), indicating the chaotic state of the liquid jet containing polymers, solvents, and nonsolvents within the high-voltage electric field.

**Figure 3:**
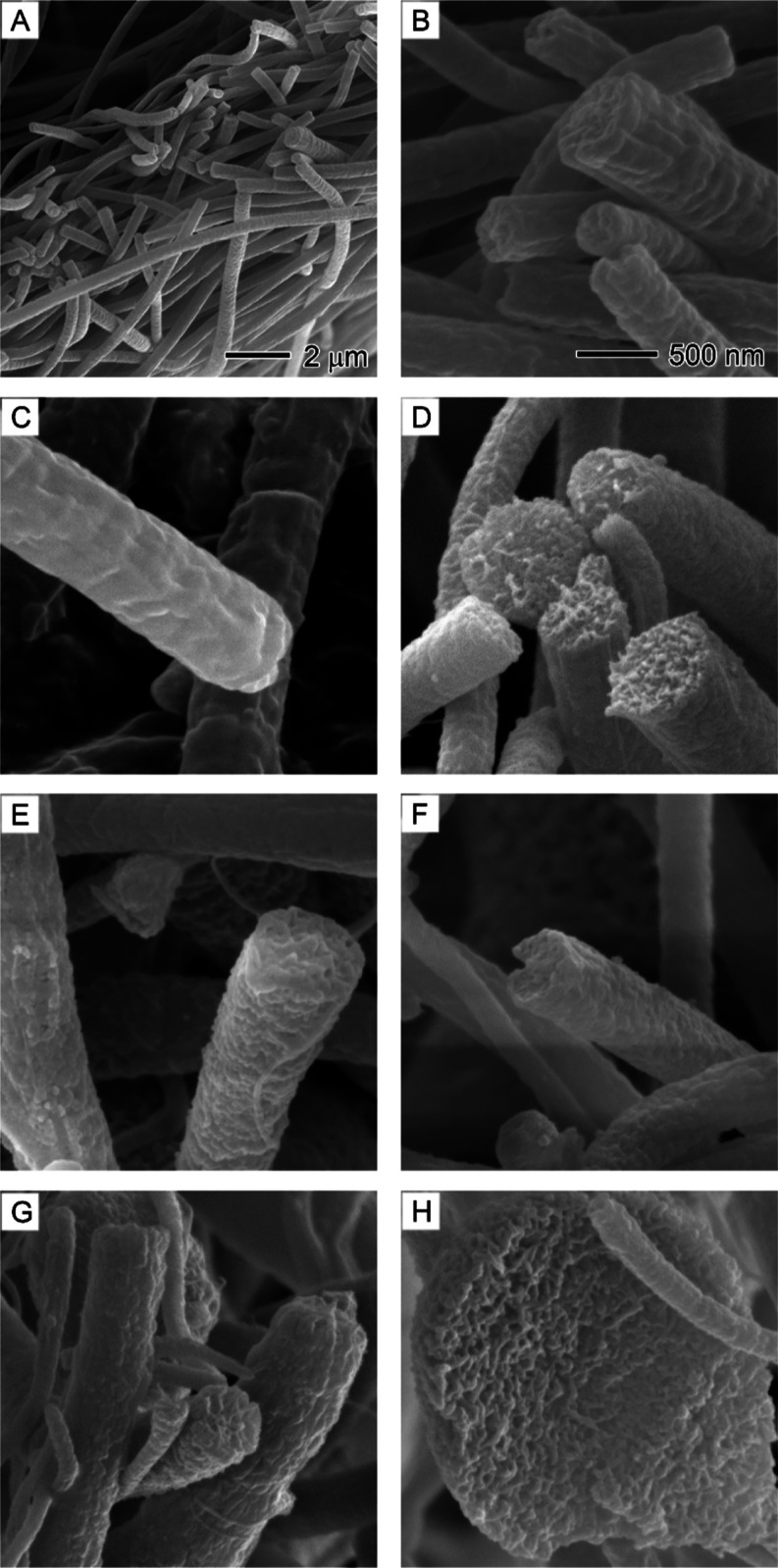
SEM images showing the surface morphology and internal structure of PAN nanofibers obtained at different core nonsolvent injection rates: (A,B) 0, (C) 0.1, (D) 0.3, (E) 0.5, (F) 0.7, and (G, H) 0.9 mL/h. The core nonsolvent is a 1/20 (v/v) water/DMF solution. The outer solution is 10% PAN in DMF. (A) is the overview of the cross-sections of PAN nanofibers. (H) is the internal structure of a bead in nanofibers. The 500 nm scale bar in (B) also applies to (C–H).

Figure 3 displays SEM images of the surface morphology and internal structure of PAN nanofibers produced at different core nonsolvent injection rates. To understand the effects of nonsolvent on nanofiber morphology, it is essential to examine the internal structure of the nanofibers before and after the introduction of nonsolvent. In the absence of nonsolvent injection, the cross-sections of all PAN nanofibers appeared solid, with no observable pores (Figure 3A,B). Although pure DMF was used as the solvent, the cooling effect of DMF evaporation might cause water from the air to condense on the surface of PAN nanofibers or diffuse into them.^26^ However, it is clear that such effects did not create any pores on the surface or the internal structure of PAN nanofibers. At a nonsolvent injection rate of 0.1 mL/h, the PAN nanofiber cross-sections exhibited an interconnected porous structure, indicative of spinodal decomposition phase separation.^37^ This nonsolvent-induced phase separation continued to expand to the surface of the nanofibers and beads, leading to the formation of rough surfaces with particle-like domains (Figure 3E–G) and honeycomb-structured beads (Figure 3H). Interestingly, no hollow channels formed even at higher nonsolvent injection rates (0.9 mL/h). This could be attributed to DMF’s higher boiling point (153 °C) compared to water (100 °C), which implies that water molecules evaporate much faster than DMF during electrospinning.^36^ Consequently, the formation of hollow channels becomes difficult due to the increasing ratio of the solvent DMF to the nonsolvent water.^38^

**Figure 4:**
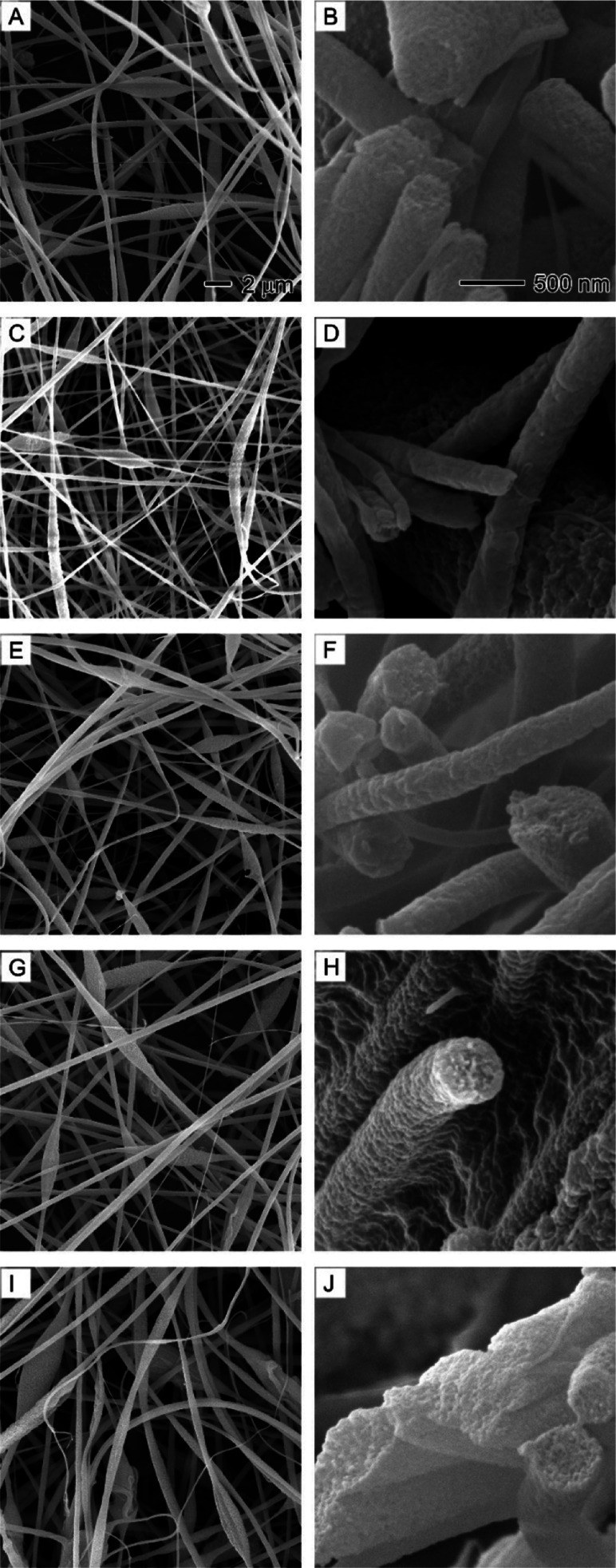
SEM images showing the effect of replacing water with ethylene glycol (EG) on the bead formation, surface morphology, and internal structure of PAN nanofibers at different nonsolvent injection rates: (A,B) 0.1, (C,D) 0.3, (E,F) 0.5, (G,H) 0.7, and (I,J) 0.9 mL/h. The core nonsolvent is a 1/20 (v/v) EG/DMF solution. The outer solution is 10% PAN in DMF. The 2 μm scale bar in (A) applies to the left column images, and the 500 nm scale in (B) applies to the right column images.

To validate our hypothesis, we selected ethylene glycol (EG) as an alternative nonsolvent, possessing a higher boiling point (197 °C) than DMF (153 °C). Like water, EG is miscible with DMF.^39^ We used a core fluid with the same percentage of nonsolvent EG and injected it into the sheath PAN fluid via coaxial electrospinning, maintaining all other parameters constant. Nanofibers were collected, ruptured in liquid N_2_, and their cross-sections were observed through SEM, as shown in Figure 4. Distinct from nanofibers obtained using water as the nonsolvent, nanofibers produced with EG as the nonsolvent exhibited fewer beads across all nonsolvent injection rates (Figure 4A,C,E,G,I). The electrospinning process was smoother with less clogging. Additionally, the liquid jet was more sensitive to airflows, which might be related to the rapid charge release from nanofibers.^40^ The discharged nanofibers were more likely to be collected on the rear collector within the fume hood, a common phenomenon for many quickly discharged polymers at higher humidity levels.^41^

**Figure 5:**
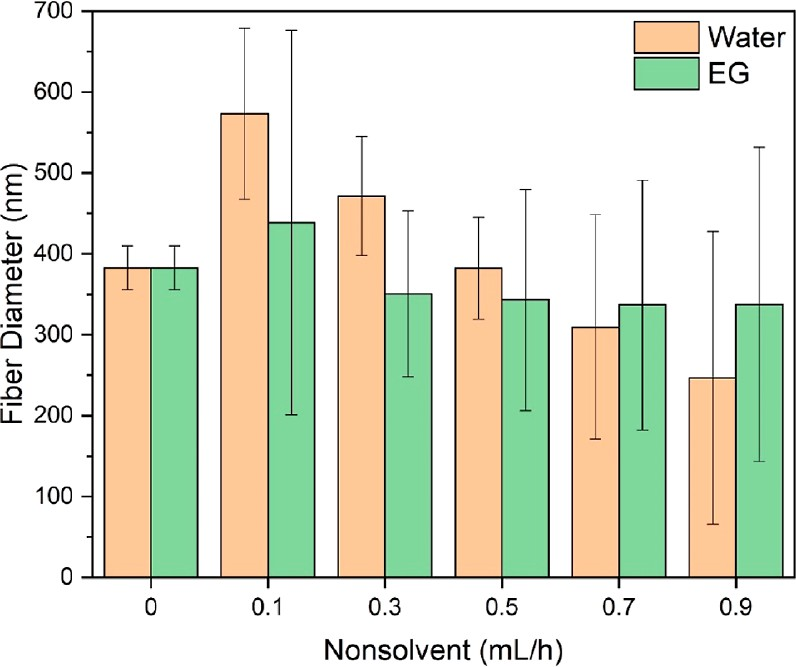
Diameters of PAN nanofibers obtained using different nonsolvent (i.e., water and EG) injection rates. The sizes of beads are excluded for accuracy.

Cross-sections revealed that all nanofibers with EG injection rates from 0.1 to 0.9 mL/h possessed porous structures similar to those generated by using water as the nonsolvent. The particulate interior appeared more pronounced for nanofibers at higher EG injection rates (Figure 4B,D,F,H,J), resulting from nonsolvent-induced phase separation. Intriguingly, we still did not observe any hollow channels in the nanofibers even at high EG injection rates (e.g., 0.9 mL/h), which deviated from our initial hypothesis. It seemed that the relative volatility of nonsolvent compared to solvent failed to fully explain the nonsolvent-induced phase separation process in generating pores inside nanofibers.^20^ Instead, intermolecular interactions between nonsolvent molecules and polymer chains appear to be key in determining whether phase separation occurs in creating porous nanofibers.^42^ Both water and EG molecules can form hydrogen bonds with the nitrile (−C≡N) groups in PAN, leading to intermolecular interactions that may induce phase separation.^43^ However, water, being a small and highly polar molecule, forms stronger hydrogen bonds due to its size and polarity, whereas EG, a larger and less polar molecule, may not induce phase separation as effectively as water in PAN solutions.^44^ Moreover, the slow evaporation of solvent DMF allowed sufficient time for the two nonsolvent molecules to penetrate polymer domains, diffuse outside, and evaporate, leaving no significant nonsolvent-rich domains inside nanofibers to form hollow channels.^45^ Our observations of nanofiber internal structures (Figures 3 and 5) aligned with the molecular interactions of the nonsolvent and polymer.

The average diameter of PAN nanofibers without nonsolvent injection was 383 nm, as shown in Figure 5. With the injection of water as the nonsolvent (0.1 mL/h), the average diameter increased by 50% to 573 nm. This increase was likely attributed to the strong interactions between the nonsolvent water and PAN.^46^ As water injection rates increased, the average nanofiber diameter continually decreased, reaching 247 nm, due to the formation of a large number of beads that significantly reduced the size of the fiber portions. Similarly, nanofibers with EG as the nonsolvent exhibited a comparable trend, with a decreased nanofiber diameter as nonsolvent injection rates increased. However, the decrease was less pronounced, ranging from 439 to 337 nm. This difference could be attributed to the weaker intermolecular interactions between EG and PAN compared to those between water and PAN.^47^

### Nonsolvent-Induced Phase Separation in PS Nanofibers

**Figure 6:**
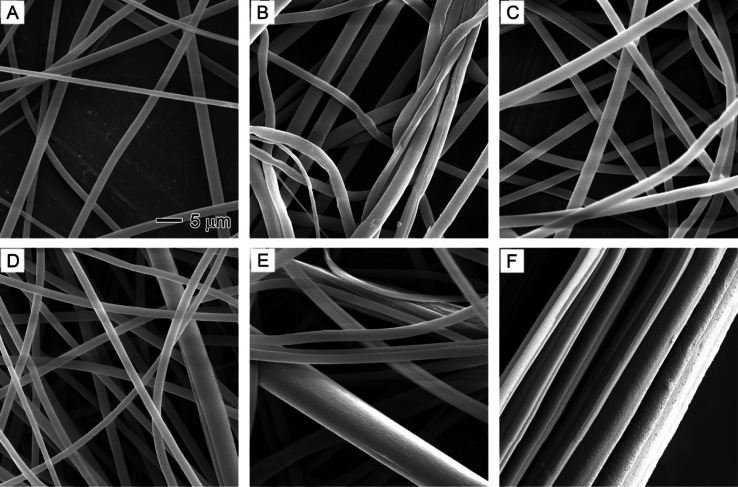
SEM images showing the effect of core nonsolvent injection rates on PS fibers: (A) 0, (B) 0.1, (C) 0.3, (D) 0.5, (E) 0.7, and (F) 0.9 mL/h. The nonsolvent is a 1/20 (v/v) water/DMF solution. The outer solution is 20% PS in DMF. The 5 μm scale bar in (A) applies to all images.

To promote phase separation, it is essential to minimize the intermolecular interactions between the nonsolvent and the polymer. Therefore, we selected polystyrene (PS) as a secondary polymer for this study. PS is a hydrophobic polymer, while water is a highly polar molecule. The intermolecular interactions between the highly polar water molecules and nonpolar PS molecules are minimal due to the hydrophobic nature of PS, which prevents significant interactions from forming with water.^48^ As a result, water acted as a plasticizer and assisted the formation of uniform fibers at various water injection rates, as depicted in Figure 6. Uniform fibers with smooth surfaces were produced at all water injection rates, corroborating our hypothesis.

**Figure 7:**
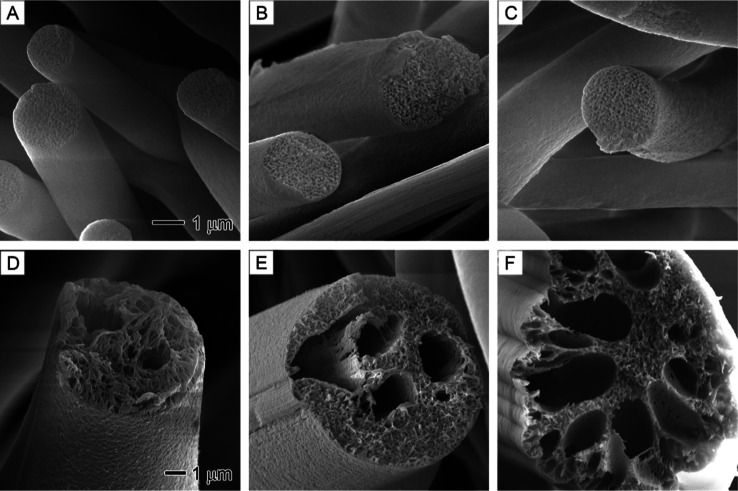
SEM images showing the internal porosity of PS fibers prepared at different core nonsolvent (1/20 v/v water/DMF) injection rates: (A) 0, (B) 0.1, (C) 0.3, (D) 0.5, (E) 0.7, and (F) 0.9 mL/h. The outer solution is 20% PS in DMF. The 1 μm scale bar in (A) applies to (A–C), and the 1 μm scale bar in (D) applies to (D–F).

As reported in our previous studies,^48−51^ PS nanofibers without the injection of water exhibited internal porosity due to the diffusion of environmental water vapor into PS, causing phase separation (Figure 7A). This phase separation was further enhanced by injecting water internally at rates of 0.1 and 0.3 mL/h (Figure 7B,C). When water injection rates were increased to above 0.5 mL/h, distinct hollow channels with interconnected pores were observed inside PS nanofibers (Figure 7D–F), which differed from the PAN nanofibers. The formation of large hollow channels resulted from the accumulation of a substantial amount of water molecules, creating water-rich domains. Owing to the hydrophobic nature of PS, the highly polar water molecules experienced minimal interactions with PS, making it less likely for large water-rich domains to effectively permeate the polymer. Consequently, hollow channels and pores emerged from the nonsolvent-rich regions. This observation underscores the significance of understanding the molecular interactions between polymers and nonsolvents to effectively manipulate phase separation and achieve the desired porous structures.

**Figure 8:**
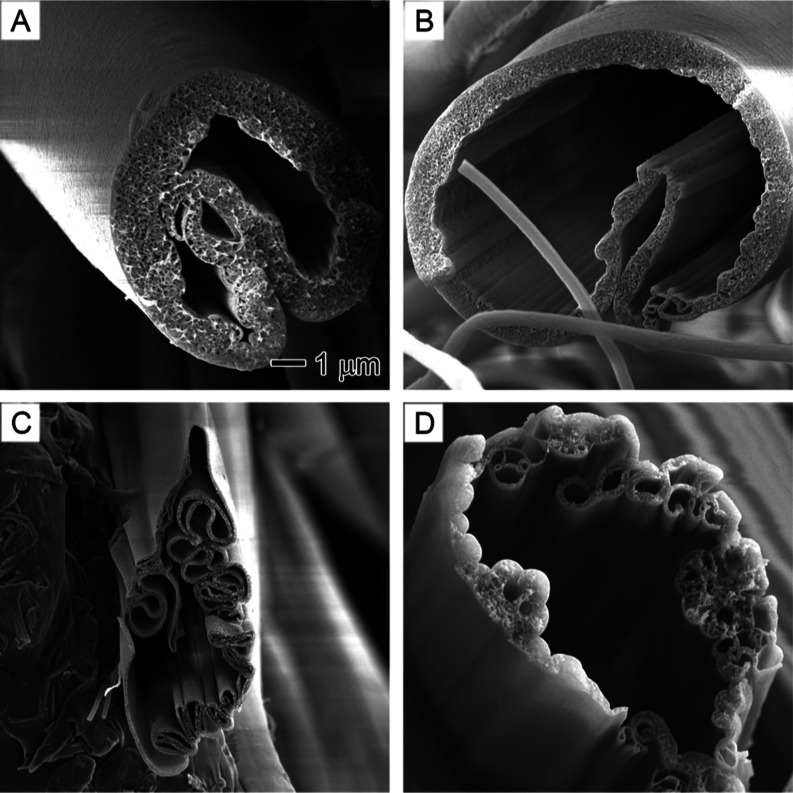
SEM images showing folding of some PS fiber sheaths due to their collapse at fast core nonsolvent (1/20 v/v water/DMF) injections: (A) 0.7, (B) 0.8, (C) 0.9, and (D) 1.0 mL/h. The outer solution is 20% PS in DMF. The 1 μm scale bar in (A) applies to all images.

The bending and whipping instability during coaxial electrospinning led to the formation of hollow fibers with unique characteristics at higher nonsolvent injection rates (0.7–1.0 mL/h), as illustrated in Figure 8. It was evident that the sheath became thinner as the water injection rate increased. Due to the formation of a large hollow channel beneath the sheath, the sheath subsequently collapsed, resulting in folded fibers (Figure 8A,B). This collapse can be attributed to the sheath’s mechanical strength being insufficient to support its own weight. In cases where the sheath was exceptionally thin and weak, hollow fibers with numerous layered folding structures were observed (Figure 8C,D).

**Figure 9:**
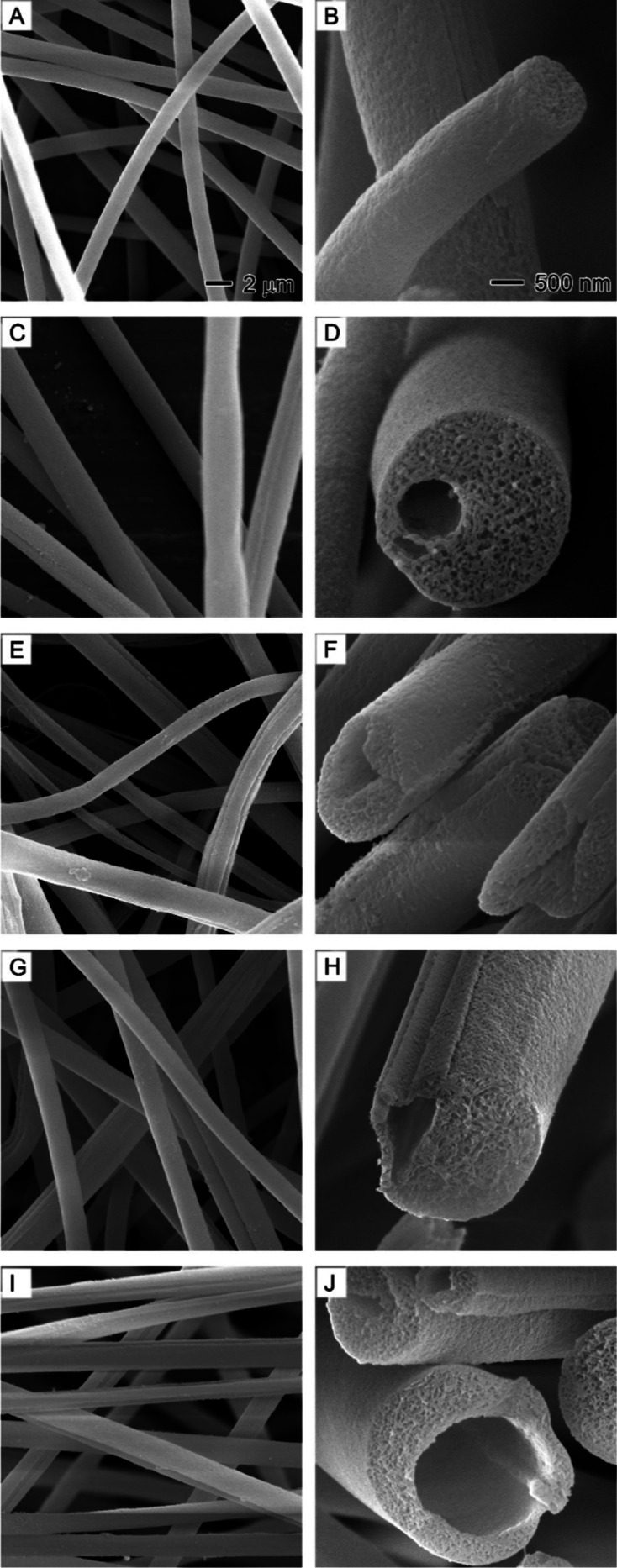
SEM images showing the effect of different core nonsolvent injection rates on the surface morphology and internal structure of PS fibers: (A,B) 0.1, (C,D) 0.3, (E,F) 0.5, (G,H) 0.7, and (I,J) 0.9 mL/h. The core nonsolvent is a 1/20 (v/v) EG/DMF solution. The outer solution is 20% PS in DMF. The 2 μm scale bar in (A) applies to the left column images, and the 500 nm scale in (B) applies to the right column images.

Figure 9 displays the surface and interior of PS nanofibers prepared at various EG injection rates. All the fibers exhibited smooth surfaces and uniform diameters, akin to fibers produced using water as a nonsolvent. As anticipated, the cross-sections revealed pore formation with the injection of EG. However, the hollow channels were relatively smaller and less pronounced compared to those produced by water. Unlike water molecules, EG exhibited a higher affinity for PS due to its capacity to form weak hydrogen bonds with the aromatic rings of PS. This characteristic might have enhanced EG’s ability to diffuse through the PS matrix. Consequently, EG is likely to have a higher permeability than water, resulting in weaker phase separation and smaller EG-rich domains. Upon drying, smaller hollow channels formed in PS, as observed in Figure 9.

**Figure 10:**
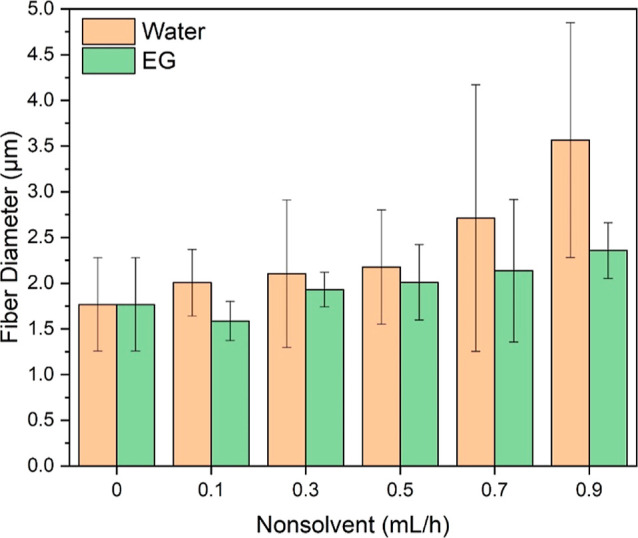
PS fiber diameters using different nonsolvent (i.e., water and EG) injection rates.

Figure 10 presents the average diameter of PS fibers using water and EG as nonsolvents. Contrary to the decreasing size trend observed for PAN nanofibers, all PS fibers exhibited an increasing trend with the rise of nonsolvent injection rates. The only exception was a slight decrease in the average size of PS fibers with a 0.1 mL/h EG injection rate from 1.77 to 1.58 μm; afterward, the size increased as the EG injection rate rose. Moreover, PS fibers generated with water had a significantly larger average size compared to those generated with EG. This difference is likely attributable to the weaker intermolecular interactions between water and PS than between EG and PS.^21^ Additionally, the lower water permeability in the PS matrix played a crucial role in determining fiber size.^52^

### Solvent Evaporation Kinetics on the Formation of Porous Structures in Nanofibers

According to the nonsolvent-induced phase separation mechanism for the formation of electrospun porous nanofibers, it is essential to maintain a polymer/solvent/nonsolvent ternary solution for a specific period to allow the process to complete effectively. If the solvent evaporates too quickly, the phase separation within the nanofibers may be insufficient, resulting in less distinct pores in the final nanofiber structure.^20^

**Figure 11:**
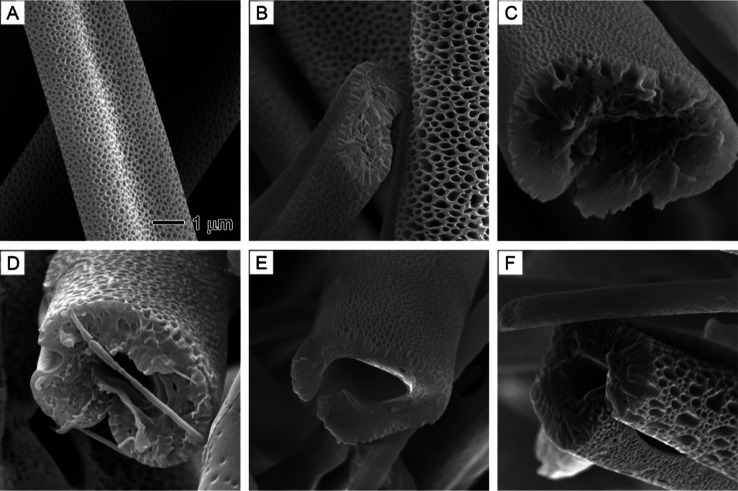
SEM images showing the surface and internal structures of PS fibers prepared by using nonsolvent (1/20 v/v water/THF) at different injection rates: (A) 0, (B) 0.1, (C) 0.3, (D) 0.5, (E) 0.7, and (F) 0.9 mL/h. The outer solution contains 20% PS in THF. The 1 μm scale bar in (A) applies to all images.

In order to investigate the influence of solvent evaporation rate on nonsolvent-induced phase separation in electrospun porous nanofibers, we replaced the solvent DMF with tetrahydrofuran (THF). THF has a lower boiling point (66 °C) than DMF (153 °C), causing it to evaporate more quickly during electrospinning. This faster evaporation could lead to a faster solidification of the fibers, potentially affecting the phase separation process. Figure 11 displays the PS fibers obtained using THF as the solvent at various water injection rates. We have previously described the formation of surface pores on PS nanofibers when using THF as the solvent.^48^ In brief, the rapid evaporation of THF resulted in the condensation of water vapor. The discharged liquid jet exhibited a strong attraction to the surrounding tiny water droplets, causing hard collisions that dented the soft surface and left shallow holes after water evaporation (Figure 11A). Due to the fast solidification of PS, electrospinning is typically unsustainable. However, injecting water into the PS solution improved the electrospinning process, likely due to the affinity between water and THF, which slowed THF evaporation. Despite this, the solidification of sheath PS occurs so rapidly that there is insufficient time for core water molecules to diffuse into the polymer solution and initiate phase separation, resulting in a solid interior or solid sheath (Figure 11B,C).

At higher water injection rates (0.5–0.9 mL/h), hollow channels were observed in PS fibers, which formed due to the accumulation of large water-rich domains. The size of these hollow channels increased slightly with higher core water injection rates, corroborating the rapid solidification of the sheath PS matrix. This observation highlights that the intermolecular interactions between nonsolvent and polymer are not the sole determining factors in the phase separation process. Instead, factors such as solvent evaporation rate and electrospinning conditions also play crucial roles in determining the final morphology of the electrospun fibers.

**Figure 12:**
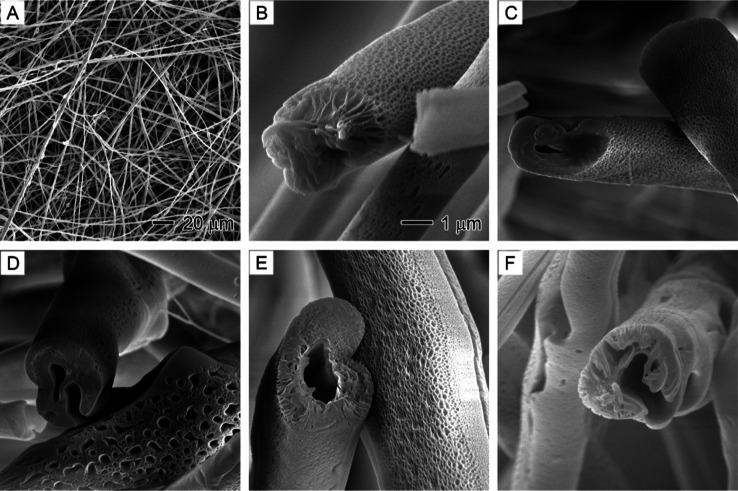
SEM images showing PS fibers prepared by using nonsolvent (1/20 v/v EG/THF) at different injection rates: (A,B) 0.1, (C) 0.3, (D) 0.5, (E) 0.7, and (F) 0.9 mL/h. The outer solution is 20% PS in THF. The 1 μm scale bar in (B) applies to (B–F).

Injecting EG into PS significantly improved the spinnability of PS when using THF as the solvent, enabling the production of a large quantity of fibers (Figure 12A). However, similar to water, EG did not induce sufficient phase separation, as evidenced by the solid nanofibers (Figure 12B) or hollow fibers with solid sheaths (Figure 12C–F). The size of hollow channels increased with the increase in EG injection rate from 0.3 to 0.9 mL/h. It is apparent that the accumulation of EG-rich domains contributed to the formation of these hollow channels. Our observations further confirmed that there was no effective phase separation in the dried polymer matrix. To initiate phase separation and the subsequent formation of internal pores, the evaporation kinetics of the solvent should be slowed down to provide an adequate amount of time for the nonsolvent to diffuse into the polymer solutions.

## Conclusions

In conclusion, this study demonstrated the critical role of molecular interactions and solvent evaporation kinetics in the formation of electrospun porous nanofibers using the coaxial electrospinning technique. By investigating the effects of water and EG as nonsolvents on both PAN and PS nanofibers, we were able to draw the following key findings: (1) The intermolecular interactions between nonsolvents and polymers played a crucial role in determining the phase separation and the formation of porous structures. Stronger interactions, such as those between water and PAN, led to less effective phase separation, while weaker interactions, such as those between water and PS, resulted in more pronounced phase separation and porous structures. (2) The size and polarity of nonsolvent molecules also influenced the phase separation process. Water, being a smaller and more polar molecule, induced more effective phase separation in PAN solutions compared to EG, which has a larger size and lower polarity. (3) The evaporation kinetics of the solvent had a significant impact on the phase separation process. Replacing DMF with a faster evaporating solvent like THF resulted in less distinct porous structures due to the rapid solidification of the fibers, leaving insufficient time for the nonsolvent to diffuse into the polymer solutions. (4) To achieve desired porous structures in electrospun nanofibers, understanding the molecular interactions between polymers and nonsolvents, as well as controlling solvent evaporation kinetics, is essential. This knowledge allows for the manipulation of phase separation processes and the creation of nanofibers with tailored properties for various applications. This work provides valuable insights into the complex relationship between molecular interactions and solvent evaporation kinetics in the electrospinning process. It offers guidance for researchers aiming to develop porous nanofibers with specific characteristics for a wide range of applications in fields such as filtration, drug delivery, and tissue engineering.
